# Human Auricles Are Not Symmetrical: A Comparative Study Using Landmark-Based and Surface-Based Software

**DOI:** 10.1097/SCS.0000000000011480

**Published:** 2025-05-20

**Authors:** Yangyang Lin, Johannes G.G. Dobbe, Nadia Lachkar, Theo H. Smit, Corstiaan C. Breugem, Geert J. Streekstra

**Affiliations:** * Department of Plastic Surgery, Renji Hospital, School of Medicine, Shanghai Jiao Tong University, Shanghai, P.R. China; † Department of Plastic, Reconstructive and Hand Surgery, Amsterdam UMC Location University of Amsterdam; ‡ Amsterdam Reproduction and Development research institute, Amsterdam, The Netherlands; § 7th Department of Plastic Surgery, Plastic Surgery Hospital, Chinese Academy of Medical Sciences and Peking Union Medical College, Beijing, China; ∥ Department of Biomedical Engineering and Physics, Amsterdam UMC location University of Amsterdam, Amsterdam; ¶ Amsterdam Movement Sciences, Musculoskeletal Health—Restoration and Development, Amsterdam; # Department of Orthopedic Surgery and Sports Medicine, Amsterdam Movement Sciences, Amsterdam UMC, University of Amsterdam, Amsterdam; ** Department of Medical Biology, Amsterdam UMC Location AMC, Amsterdam; †† Department of Gynaecology and Amsterdam Reproduction and Development, Amsterdam UMC Location VUmc, Amsterdam, The Netherlands

**Keywords:** Auricle symmetry, automated measurement, bilateral differences, ear reconstruction, high-precision technique, manual measurement, surface-based measurement

## Abstract

**Purpose:**

To evaluate the outcome of ear reconstruction, the contralateral auricle is often used as a reference. Literature has shown that auricles are symmetric on a group level, but it is unknown if this is also true on an individual level. In this paper, the authors quantify bilateral symmetry of the auricle and hypothesize that quantifying bilateral differences on an individual level requires a technique that is more precise than conventional manual measurement methods.

**Methods:**

CT sinus scans of 42 healthy volunteers were used to determine the bilateral symmetry of the auricle using conventional manual measurement techniques and a high-precision computer-assisted surface-based technique with a high precision of measurement. Bilateral symmetry was evaluated for the following geometric auricle parameters: length, width, protrusion distance, auriculocephalic angle, inclination angle, posteroanterior and superoinferior position difference. Bilateral differences exceeding the reliability threshold, based on the precision of measurement for the manual and automated approaches, were considered true differences.

**Results:**

In both the manual and automated measurements, the authors found no statistically significant differences in bilateral auricle parameters at the group level when evaluating a group of L versus R ears. At the individual level, however, the automated algorithm established bilateral differences in auricle parameters in 74% to 100% of the cases, whereas manual measurements could only detect bilateral differences in 7% to 64% of the cases.

**Conclusions:**

The high-precision surface-based technique shows that human auricles are generally not symmetric on the individual level. Future research is needed to investigate which discrepancies are acceptable from a technical point of view and which left-right differences are considered acceptable from a cosmetic and reconstructive point of view.

About 5% of the general population has protruding ears.^1^ Many of these patients request surgery later in life, making otoplasty a relatively common surgical procedure. On the other hand, microtia is a rather uncommon malformation, with an incidence of about 0.83 to 17.4 per 10,000 live births.^2^ Microtia ear reconstruction and otoplasty are intricate surgical procedures. Achieving a high level of symmetry is usually presumed essential for patient satisfaction.^3^ In preoperative planning and postoperative evaluation, physicians frequently utilize the unaffected contralateral side as a reference standard, hereby implicitly assuming symmetry between both auricles. Key esthetic and therapeutic parameters for auricle reconstruction include auricular length, width, protrusion, auriculocephalic angle, auricular inclination, and bilateral position.

In the literature, the conclusions regarding auricle symmetry vary for the different geometric parameters. Length, width, area, and ratio in general show good symmetry between the left and right ears, while angular dimensions tend to be asymmetric. In a study by Sforza et al,^4^ for example, no significant differences were found in ear length and width, but the auriculocephalic angle was smaller in the right ear when measured physically by rulers. Fu et al^5^ reported symmetry in ear length, width, and protrusion using 3D landmarking methods, but Tatlisumak et al found asymmetry in ear length, width, and protrusion using 2D Photos.^6^

An explanation for the different observations in these studies may be the variability in precision between the utilized measurement methods. The key point here is that a variability in the measurement of an auricle parameter, which is larger than the bilateral difference, will make it impossible to establish a bilateral difference in that parameter and will therefore render it “symmetric.” In this context, direct physical and photo measurements have been proven to be relatively imprecise.^7–10^ Several studies suggest that 3D landmarking methods can enhance precision.^11–14^ Nevertheless, these methods still rely on skilled anthropometrists, leading to potential measurement variability and limited objectivity.^15^

To be optimally sensitive in detecting small bilateral differences, we have published a precise method for measuring auricle parameters.^16^ Applying this high-precision measurement algorithm to a CT database of auricles without notable malformations may offer more information on auricle symmetry than traditional manual measurement approaches. Hence, we hypothesize that high-precision automatic measurement methods can reveal bilateral differences in auricle parameters at the individual level that cannot be determined with less precise manual methods.

## METHODS

**FIGURE 1:**
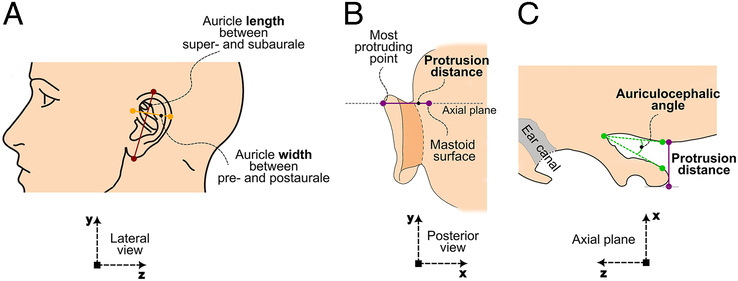
Manual 3D measurements. (A) Lateral view of the head, showing length (red), width (yellow). (B) Posterior view of the head, showing protrusion distance (purple) and the place to axial plane (gray). (C) Axial plane view of the head, showing auriculocephalic angle (green) and protrusion distance (purple). The coordinating systems are in line with those in Figure 2.

This study compares 2 techniques to measure a number of auricle parameters: manually, supported by 3D landmarking software,^12^ and a novel semiautomatic surface-based approach.^16^ Both techniques use virtual auricle models derived from CT image data. Both approaches provide commonly used auricle parameters according to clinical practice at the Amsterdam University Medical Centers and are also used in auricle-reconstruction literature, ie, auricle length, width, protrusion distance, auriculocephalic angle, inclination angle, and position (Fig. 1 and Supplemental Table 1, Supplemental Digital Content 1, http://links.lww.com/SCS/H865) The method to obtain virtual head models from CT images by segmentation, and the approach to derive auricle parameters from these mesh models is described in detail in a recent paper by our group^16^ and will be described shortly in the following sections.

### Auricle Data and CT Image Acquisition

One hundred CT head scans of patients who underwent CT sinus scanning were retrieved from the image archiving system. The CT scans were made on regular clinical CT scanners with pixel dimensions varying between 0.34 and 0.46 mm and slice thickness between 1 and 1.5 mm. The scans were included in the study if they showed both auricles. Scans were excluded if the slice thickness was 2 mm or higher; auricles were not fully visible in the scan due to positioning or partial imaging; auricles were not free in de scan, for example, deformed due to the use of a pillow or head rest during scanning; an auricle was distorted due to trauma, surgery or congenital anomalies. After screening, 42 scans remained for the evaluation of bilateral symmetry in this study.

### Virtual Head Models

Virtual models of the auricles were obtained by segmenting the head from the sinus scan and subsequently extracting the auricles. Segmentation is based on a Laplacian level-set segmentation algorithm,^17^ which is initialized by a region growing and subsequent filling algorithm to close gaps inside the object and to close the outline. The Marching cubes algorithm^18^ is used to extract a polygon mesh at the zero-level of the level-set image.

### Conventional Approach to Measure Auricle Parameters

One of the authors (YL) utilized the 3D landmarking technique within MIMICs software (Materialise, Leuven, Belgium) to assess the auricle parameters of both ears. This method allows the user to select specific points on the 3D model of a virtual head, which are used for calculating distances and angles. The auricle parameters that are measured are listed in Table 1, Supplemental Digital Content 1, http://links.lww.com/SCS/H865, and are illustrated in Figure 1.

### Semiautomatic Approach to Measure Auricle Parameters

**FIGURE 2:**
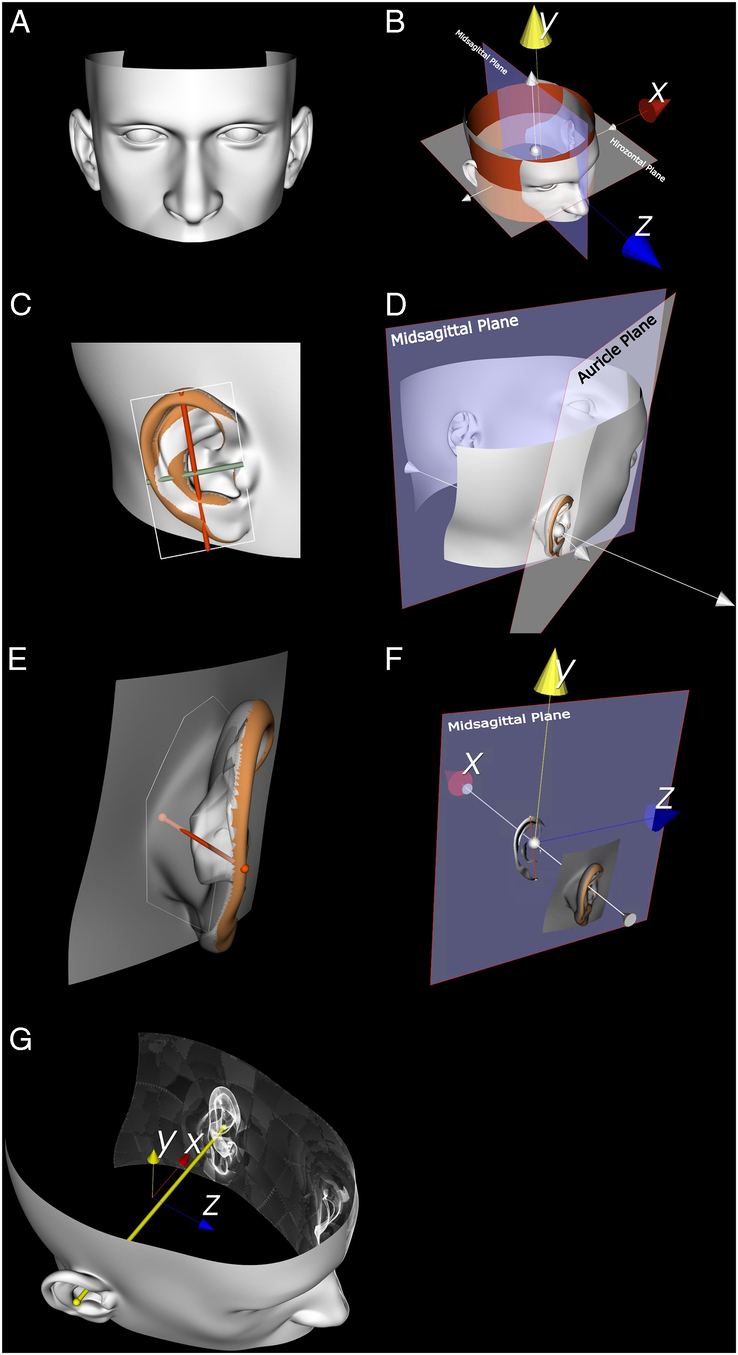
This set of images (labeled A–G) illustrates the automated process of auricle measurement using a 3D head model constructed from CT scans. The auricle surfaces are marked in orange, and the head model is marked in white. (A) The creation of a 3D head model is displayed. (B) The blue plane represents the midsagittal plane identified by a symmetry algorithm, determining the *x*-axis. Simultaneously, the white plane signifies the horizontal plane established by a cylinder algorithm (red cylinder), which determines the *y*-axis. The *z*-axis is subsequently defined by the orthogonal relationship to the *x* and *y*-axes. (C) Auricle length (depicted by the red line) and width (depicted by the green line) are measured using a principal component algorithm(white box). (D) A white plane is fitted to the auricle surface, and the auriculocephalic angle is determined by the angle between this white plane and the midsagittal plane (blue plane). (E) The mastoid plane(white ring) is situated in the mastoid region behind the ear. Auricle protrusion (red line) is calculated as the maximum distance from the auricle surface perpendicular to the mastoid plane. (F) The auricle surface is projected onto the midsagittal plane, resulting in a flattened auricle shape(white, projected from the left ear as an example). The auricle axis (orange line) is then derived from this flat shape and extended to intersect with the *y*-axis (yellow line). (G) A yellow line connects the bilateral centroids of the auricles. This line is projected onto the *y-* and *z-*axes, respectively, to calculate the superoinferior and posteroanterior disparities of the bilateral auricles.

The novel semiautomatic and surface-based approach is described in detail in the study by Liu et al^16^ (Fig. 2) (Supplemental Table 1, Supplemental Digital Content 1, http://links.lww.com/SCS/H865). In short, first, the most lateral surface of each auricle is selected. Next, a plane is fit through these points, and the points are subsequently projected in that plane. Then, principal component analysis (PCA) on the points in each auricle mesh is used to find the inertial axes in length and width. Projecting all points in an auricle mesh onto these axes and taking the distance between the outermost points provides the auricle length and width. In addition, points on the mastoid surface of the head are selected, lying behind the auricle surface. Planes are fit to these points on the mastoid surface and to the auricle surface, and the angle between the plane normal vectors, ie, the vectors perpendicular to these planes, defines the auriculocephalic angle. The protrusion distance is the largest distance between the mastoid plane to any point in the auricle surface, and is measured perpendicular to the mastoid plane.

Quantifying the bilateral difference in posteroanterior and inferosuperior auricle position, and the auricle inclination requires defining a local coordinate system. For this purpose, the *x*- and *y*-axes are found by determining the plane of symmetry of the head where the plane normal vector defines the direction of the +*x*-axis, and fitting a cylinder to the head where the central axis defines the direction of the +*y*-axis. The +*z*-axis is perpendicular to the +*x*- and +*y*-axes according to the right-hand rule. The centroid of our head model is projected in the plane of symmetry and defines the origin of our coordinate system. The inclination angle is the angle between the length axis of the auricle and the +*y*-axis of the coordinate system. The centroid of each auricle serves as the position of that auricle, and the bilateral difference in auricle position is found by projecting these points onto the +*y*- and +*z*-axes to find, respectively, the inferoposterior position and the posteroanterior position.

### Ability of Measuring Bilateral Symmetry of Auricle Parameters

The absolute difference between the left and right of an auricle parameter was calculated to assess bilateral symmetry. A reliability threshold for bilateral difference is calculated for each parameter to allow for making a statement about the percentage of cases where the bilateral difference is statistically significantly different from zero at the 5% confidence level.

Each reliability threshold is calculated utilizing a precision measure in terms of an SD as presented in our previous paper.^16^ In that paper, the precision was obtained by repeated scans of a single cadaver head and subsequent quantification of auricle parameters. To arrive at a reliability threshold *T*_*rel*_ for bilateral differences, we used the following relationship:^19^


(1) $$T_{\mathrm{rel}}=1.96*\mathrm{SD}*\sqrt{2(1-r)}$$

where *r* is the correlation coefficient of the linear trend line between the left and the right values of the auricle parameter.

It is important to note that there are no manual reliability thresholds for inclination angle, posteroanterior, and superoinferior difference since measuring these parameters manually is fairly impossible. The automatic posteroanterior and superoinferior difference parameters already represent a difference between auricle positions. The reliability threshold for these parameters are therefore 1.96*SD.

For comparison with findings in literature, we calculated the average and SD and performed *t* tests on the values of the left and right auricle for each parameter and for each method. All analyses were performed using R Studio (RStudio 2022.02.3+492 “Prairie Trillium” Release).

## RESULTS

### Reliability Threshold in Automatic and Manual Methods

For length, width, protrusion, and auriculocephalic angle, manual methods have significantly higher reliability thresholds compared with the automatic approach (Supplemental Table 2, Supplemental Digital Content 1, http://links.lww.com/SCS/H865), rendering the manual method less discriminative in assessing left-right differences. The inclination angle and positional differences were not assessable manually, further highlighting the advantages of automatic methods in comprehensive auricle evaluation.

### Ability of Measuring Bilateral Symmetry of Auricle Parameters at the Individual Level

**FIGURE 3:**
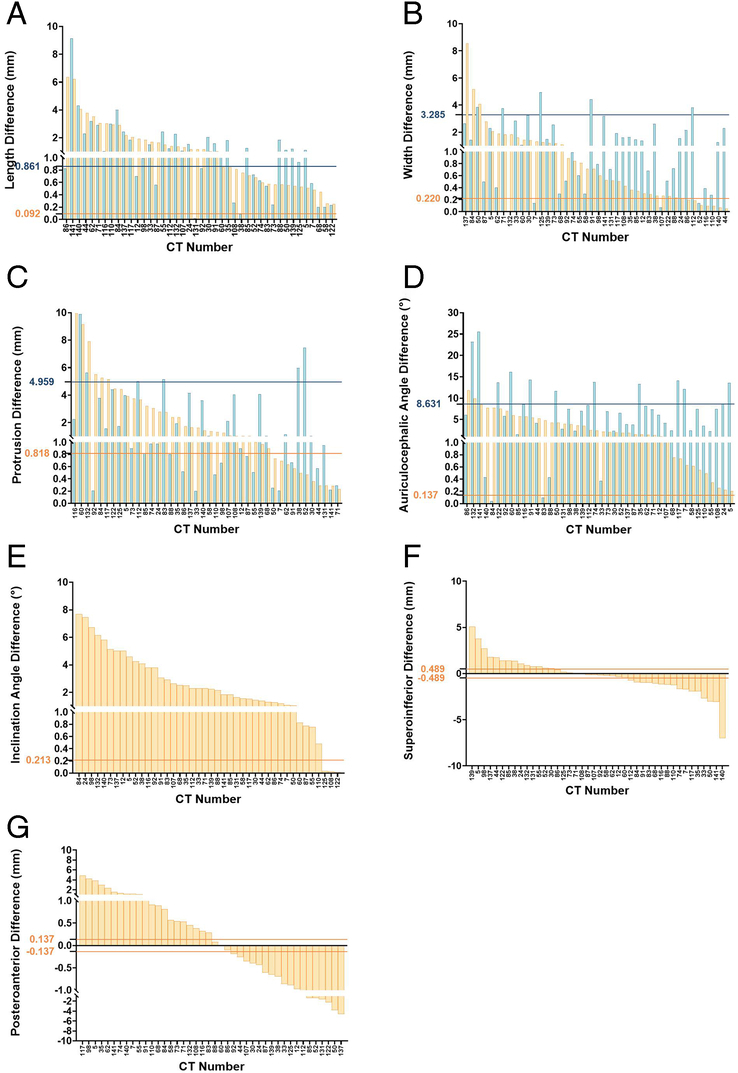
Bilateral asymmetry in auricle parameters, showing differences in length (A), width (B), protrusion distance (C), auriculocephalic angle (D), inclination angle (E), superoinferior position (F), posteroanterior position (G). See Supplemental Table 1, Supplemental Digital Content 1, http://links.lww.com/SCS/H865, for definitions of these parameters. The horizontal lines represent confidence thresholds for the conventional manual measurement approach (blue) and for the surface-based high-precision method (orange). Bilateral differences higher than these thresholds are considered true bilateral differences. No manual data were provided for inclination angle and positional difference measurement (E–G).

As the reliability thresholds for automatic measurements are much lower than those of their manual counterparts, automatic methods were able to discern more auricular asymmetry at the individual level than manual measurements (Fig. 3). For ear length, asymmetry was detected by the automated algorithm in 100% of subjects, with the highest and lowest difference being 6.36 and 0.25 mm. Manual measurements only found length asymmetry in 62% of individuals. For ear width, 83% of subjects were found to have asymmetry by the automated algorithm, with the highest difference being 8.53 mm, while only 17% were found to have asymmetry by manual measurement. The automatic algorithm could discern asymmetry in ear protrusion for 74% of cases, with the highest difference measured at 9.94 mm, while only 7% of asymmetry could be detected by manual measurement. The automated algorithm could discern 100% of auriculocephalic angle asymmetry, reaching up to 11.8 degrees. The automated algorithm was also able to discern a bilateral difference in 93% of inclination angles. However, due to the lack of a manual measurement method, a comparison could not be made (Supplemental Table 3, Supplemental Digital Content 1, http://links.lww.com/SCS/H865) on an Individual level.

### Bilateral Symmetry of Auricle Parameters at the Group Level

For manual measurements, the length, width, protrusion, and auriculocephalic angle showed no significant deviation from normality (*P*>0.05), suggesting a normal distribution across these parameters. Automatic measurements echoed these findings, with all parameters, including the inclination angle and position differences, displaying a normal distribution, except for the superoinferior position (*P* = 0.01145), indicating potential non-normality in this specific measurement (Supplemental Table 3, Supplemental Digital Content 1, http://links.lww.com/SCS/H865).

Comparing left and right auricle parameters at the group level, both manual and automatic methods showed no significant bilateral differences across all parameters when evaluating a group of L versu R ears, with *P*-values >0.05, suggesting symmetry at the group level. (Supplemental Table 3, Supplemental Digital Content 1, http://links.lww.com/SCS/H865, on a group level).

## DISCUSSION

In auricle reconstruction, the contralateral side usually serves as a reference, since no statistically significant left-right differences are reported on a group level.^5,20–22^ Although facial symmetry is considered more attractive,^23^ it was unknown what the level of asymmetry is in a sample of individuals with normal auricles. In this study, we investigated bilateral symmetry of the auricle at an individual level using traditional manual approaches with a standard precision of measurement, versus a computer-assisted surface-based approach with a high precision of measurement. It was found that for the majority of cases in our study population bilateral auricle differences exceeded our reliability threshold for most geometric measurement parameters when measured by a more precise automatic measurement technique, hereby indicating that true differences were observed in 71% of the cases for the superoinferior position, up to 100% for length measurements, of the individuals. When using the traditional manual approaches, the reliability threshold was much higher, which rendered establishing true bilateral differences impossible for 38% to 93% of the individuals.

Linear parameters, such as length, width, and protrusion distance, have been widely measured by previous studies using various measurement techniques, including 3D landmarking^5,20,21^ and caliper measurements.^22^ Their results have generally suggested that linear parameters do not show significant bilateral asymmetry at the group level. These findings are in line with our observation that no significant bilateral differences are seen at the group level in either method. Apparently, the variability of the mean of the left and right auricle parameters is relatively high due to interindividual size differences. This renders finding bilateral differences at the group level impossible, even though our technique is much more precise than traditional approaches.

Previous attempts to quantify auricular angles, like the inclination angle and the auriculocephalic angle, have encountered methodological difficulties, leading to limited and sometimes unreliable data. Despite being identified as critical parameters in auricular reconstruction and otoplasty, systematic validation of their value in a normal population has been rare, with existing studies often concluding that these angles demonstrate bilateral symmetry.^20^ In contrast, our study, employing high-precision algorithms, has identified notable asymmetry in the auriculocephalic angle at the individual level.

It has always been a great challenge determining how much an ear protrudes. This is important for the indication of possible protruding ear surgery, but also to determine the success of the surgical procedure. Moreover, measuring ear protrusion after microtia reconstruction is also prudent to evaluate the surgical outcome. In Figure 2, we explained the new novel way to determine the auriculo-cephalic angle. This automated method could create an easy way to help surgeons determine candidates for possible surgical procedures and help us evaluate possible techniques and patient satisfaction.

In this study, we used sinus scans of a random population from the institute’s archiving system, which may have included patients with deviating ears. This may have biased the observation of normal left-right differences. However, the main goal of our study was to show that intra-individual auricle differences exist, and for that purpose, the data set was appropriate. For future study, we recommend including more individuals to find a left-right difference in a larger population. We further recommend performing future research to find what differences can be considered acceptable from a cosmetic point of view, which is of interest to take into account for reconstructive surgery.

Our study has demonstrated that individual left and right auricles are asymmetric, and that the asymmetry cannot be observed by comparing the average of left and right auricle parameters of a group of individuals. This finding challenges the conventional surgical pursuit of perfect symmetry in auricular reconstruction, advocating for a more nuanced and realistic approach. Our research suggests adopting a more individualized error margin in reconstructive surgeries, potentially enhancing the esthetic and functional outcomes of such procedures. This study not only advances our understanding of auricular anatomy but may also have profound implications for future clinical practices and patient satisfaction in facial reconstructive surgery.

## Supplementary Material
