# Supplementary material for: Human Auricles Are Not Symmetrical: A Comparative Study Using Landmark-Based and Surface-Based Software
Source: J Craniofac Surg. 2025 May 20;36(7):2559–63. doi: 10.1097/SCS.0000000000011480 (PMC12502933; doi:10.1097/SCS.0000000000011480)
Supplement: Supplementary file 1 [file scs-36-02559-s001.docx]

**Supplemental Table 1** **Auricle parameters as measured in this study by the landmarking using virtual head models and 3D software and the novel surface-based and automated approach using virtual head models**

| **Auricle parameter** | **Landmarking** | **Quick reference of Manual approach** | **Automatic** | **Quick reference of Automatic approach** |
| --- | --- | --- | --- | --- |
| AC angle | ☑️ | The angle between the back side of the auricle and the mastoid at the point where a horizontal line through the otobasion superius meets the helix | ☑️ | Angle between the normal vectors of the plane fit through the auricle surface and the mid-sagittal plane |
| Protrusion distance | ☑️ | Perpendicular distance from the most lateral (prominent) point of the ear to the mastoid | ☑️ | Distance between most-lateral auricle point and plane fit through mastoid surface |
| Length | ☑️ | Distance from the Supaurale to the Subaurale | ☑️ | Longest axis after PCA. |
| Width | ☑️ | Distance from the Preaurale to the Postaurale | ☑️ | Second longest axis after PCA |
| Inclination angle |  |  | ☑️ | Angle between the longest axis after PCA and the vertical axis of the CS |
| Posterior-Anterior Position |  |  | ☑️ | Distance(z axis, see Fig. 1c) between center positions of the left and right auricle, projected in the mid-sagittal plane. |
| Superior-inferior Position |  |  | ☑️ | Distance(y axis, see Fig. 1c) between center positions of the left and right auricle, projected in the mid-sagittal plane. |

PCA = Principal Component Analysis

CS = Coordinate system

**Supplemental Table 2 Reliability threshold in Automatic and Manual Methods**

|  | Automatic | Manual | |
| --- | --- | --- | --- |
| Length | 0.092 | 0.861 | |
| Width | 0.220 | 3.285 | |
| Protrusion | 0.818 | 4.959 | |
| Auriculocephalic Angle | 0.137 | 8.631 | |
| Inclination Angle | 0.213 | NA | |
| Posteroanterior Difference | 0.137 | NA |  |
| Superoinferior Difference | 0.489 | NA |  |

**Supplemental Table 3 Bilateral differences at a group and individual level**

|  |  | At group level | | | At individual level |
| --- | --- | --- | --- | --- | --- |
|  | 1. Parameters | Left | Right | Left vs Right | Asymmetrical confirmed |
|  |  | Mean±SD | Mean±SD | P values | Percentages |
| Manual | Length (mm) | 65.3±5.7 | 65.3±4.7 | 1.0 | 62% |
|  | Width (mm) | 37.1±3.8 | 36.7±4.0 | 0.6 | 17% |
|  | Protrusion (mm) | 17.3±3.1 | 17.1±2.7 | 0.7 | 7% |
|  | Auriculocephalic Angle (°) | 67.2±11.1 | 66.8±13.8 | 0.9 | 26% |
| Automatic | Length (mm) | 65.3±5.7 | 65.7±5.0 | 0.7 | 100% |
|  | Width (mm) | 36.3±3.6 | 36.8±4.5 | 0.6 | 83% |
|  | Protrusion (mm) | 17.9±3.7 | 17.1±4.6 | 0.4 | 74% |
|  | Auriculocephalic Angle (°) | 18.2±7.1 | 16.6±7.4 | 0.3 | 100% |
|  | Inclination Angle (°) | 5.3±6.8 | 6.9±6.4 | 0.3 | 93% |
|  | Posteroanterior Position (mm) | NA | NA | NA | 95% |
|  | Superoinferior Position (mm) | NA | NA | NA | 71% |

Table 3 summarizes the comparison of auricle parameters measured manually and automatically at both group and individual levels. It details mean values with standard deviation (Mean±SD) for each parameter on the left and right sides and the p-values indicating statistical significance of left vs right by two-sample t-test. Percentage of individuals showing asymmetry confirmed by manual(blue) and automatic(orange) measurements.
